# Primary infection of BALB/c mice with a dengue virus type 4 strain leads to kidney injury

**DOI:** 10.1590/0074-02760220255

**Published:** 2023-05-08

**Authors:** Arthur da Costa Rasinhas, Fernanda Cunha Jácome, Gabriela Cardoso Caldas, Ana Luisa Teixeira de Almeida, Daniel Dias Coutinho de Souza, João Paulo Rodrigues dos Santos, Helver Gonçalves Dias, Eduarda Lima Araujo, Ronaldo Mohana-Borges, Ortrud Monika Barth, Flavia Barreto dos Santos, Debora Ferreira Barreto-Vieira

**Affiliations:** 1 Fundação Oswaldo Cruz-Fiocruz, Instituto Oswaldo Cruz, Laboratório de Morfologia e Morfogênese Viral, Rio de Janeiro, RJ, Brasil; 2 Fundação Oswaldo Cruz-Fiocruz, Instituto Oswaldo Cruz, Laboratório de Imunologia Viral, Rio de Janeiro, RJ, Brasil; 3 Fundação Oswaldo Cruz-Fiocruz, Instituto Oswaldo Cruz, Laboratório de Patologia, Rio de Janeiro, RJ, Brasil; 4 Universidade Federal do Rio de Janeiro, Instituto de Biofísica Carlos Chagas Filho, Laboratório de Biotecnologia e Bioengenharia Estrutural, Rio de Janeiro, RJ, Brasil

**Keywords:** DENV-4, BALB/c mice, kidney, histopathology, ultrastructure

## Abstract

**BACKGROUND:**

Dengue is a disease caused by dengue virus (DENV-1 through -4). Among the four serotypes, DENV-4 remains the least studied. Acute kidney injury is a potential complication of dengue generally associated with severe dengue infection.

**OBJECTIVES:**

The goal of this study was to investigate the alterations caused by experimental dengue infection in the kidney of adult BALB/c mice.

**METHODS:**

In this study, BALB/c mice were infected through the intravenous route with a DENV-4 strain, isolated from a human patient. The kidneys of the mice were procured and subject to histopathological and ultrastructural analysis.

**FINDINGS:**

The presence of the viral antigen was confirmed through immunohistochemistry. Analysis of tissue sections revealed the presence of inflammatory cell infiltrate throughout the parenchyma. Glomerular enlargement was a common find. Necrosis of tubular cells and haemorrhage were also observed. Analysis of the kidney on a transmission electron microscope allowed a closer look into the necrotic tubular cells, which presented nuclei with condensed chromatin, and loss of cytoplasm.

**MAIN CONCLUSIONS:**

Even though the kidney is probably not a primary target of dengue infection in mice, the inoculation of the virus in the blood appears to damage the renal tissue through local inflammation.

Dengue is a tropical febrile disease transmitted by mosquitoes of the *Aedes* genus and caused by each of the four serotypes of the dengue virus (DENV-1,-2,-3 and-4).^1^ According to the World Health Organization (WHO), over 390 million infections occur every year, with 3.9 billion people living in areas with risk of transmission. The four serotypes circulate simultaneously in many countries, and, as of 2010, Brazil is listed among these countries, due to the reintroduction of DENV-4.^2^ ^,^ ^3^ While most cases of dengue remain mild or asymptomatic, one in twenty cases evolve to what is called severe dengue (SD), a condition closely associated with intense haemorrhage, plasma leakage and multiple organ impairment.^4^ ^,^ ^5^ Even though the liver is the most commonly affected organ,^6^ ^,^ ^7^ DENV has been shown to infect a wide range of biological tissues, such as the lungs,^8^ the heart,^9^ the brain^10^ and even the kidneys,^11^ among others.

Thus far, the alterations caused by DENV infection in the kidney remain largely unexplored, despite reports of signs and symptoms such as proteinuria, haematuria, glomerulonephritis, nephrotic syndrome and elevation of serum creatinine levels.^12^ ^,^ ^13^ ^,^ ^14^ More severe manifestations, such as acute kidney injury (AKI), rhabdomyolysis, glomerulonephritis, haemolytic uremic syndrome, and acute renal failure, are often associated to lethal cases of SD.^11^ ^,^ ^15^ ^,^ ^16^ Histopathological alterations described in renal tissue include congestion of the glomerular capillary, focal haemorrhage, oedema, inflammatory cell infiltration, hydropic degeneration, formation of micro abscesses, thrombus formation in the glomeruli, glomerular congestion and acute necrosis of proximal and distal tubules.^6^ ^,^ ^17^ ^-^ ^22^ On an ultrastructural level, these necrotic tubular cells were shown to be undergoing pyknosis, with dilated endoplasmic reticulum.^6^ DENV like particles have already been directly observed in the kidney through transmission electron microscopy, suggesting viral infection.^17^ Viral antigens have also been previously detected in the kidney, in tubular cells,^23^ in inflammatory cells,^24^ in glomerular endothelial cells, in mesangial cells,^21^ ^,^ ^22^ in hematopoietic cells^25^ and in circulating macrophages and monocytes.^6^ Additionally, the detection of DENV RNA through molecular technique has been reported in kidney.^8^

AKI is a renal manifestation of dengue that is often reported in the literature. This condition is characterised by a sudden decrease of kidney function, which can culminate in death.^12^ The severity of dengue increases the risk of AKI, with it being reported in 11.8% of patients with dengue with warning signs and in 28.6% of patients with SD.^26^ Around 10 to 20% of patients presenting dengue-induced AKI may require dialysis following the resolution of the disease.^27^ Despite this, dengue-induced AKI remains a poorly explored manifestation.^28^ ^,^ ^29^ ^,^ ^30^ The histopathology of AKI is characterised by proximal and distal tubule necrosis, with lumen dilation, loss of the brush border, simplification of the tubular lining epithelium, and loss of nuclei.^31^ Although the mechanisms that lead to AKI are not yet fully understood, its appearance is hypothesised to be due to a series of factors, such as the direct cytopathic viral action, cytokine induced haemodynamical alterations, deposition of antigen-antibody immune complexes, rhabdomyolysis, haemolysis and acute glomerular injury.^13^ ^,^ ^16^ ^,^ ^27^ ^,^ ^32^

Over the years, the BALB/c mouse has proven itself to be a useful animal model for dengue infection studies, replicating many aspects of the disease as it manifests in human cases. These animals not only present immune response against DENV, but also manifest histopathological alterations in liver, lung, heart, kidney, brain, spleen and skeletal muscle.^33^ ^-^ ^43^ Furthermore, currently, DENV-4 remains the least studied serotype of DENV, with most studies focusing on serotype 2.^40^ Since DENV-4 is known to cause milder cases of dengue,^44^ it is unclear whether the kidney is a target organ for infection. While few studies focus on the renal manifestations caused by dengue in BALB/c mice, alterations such as an increase in glomerular volume and in mesangial cellularity, inflammatory cell infiltration, peritubular congestion, tubular necrosis, loss of brush border microvilli, cytoplasmic loss, glomerular atrophy and focal haemorrhage have been previously reported.^45^ ^,^ ^46^ The viral antigen has also been successfully detected in DENV-infected mouse kidney, in tubular epithelial cells and in endothelial cells.^46^

Given the scarcity of data on AKI in humans and the lack of histopathological studies on kidney, this study aims to investigate the renal manifestations of dengue in BALB/c mice infected with DENV-4.

## MATERIALS AND METHODS

*Ethics statement* - All the procedures performed during the course of this study were in compliance with the principles and regulations stablished by the Brazilian College of Animal Experimentation and previously approved by the Animal Ethics Committee of Instituto Oswaldo Cruz (IOC), Fundação Oswaldo Cruz (Fiocruz), under protocol number L-023/2018.

*Viral strain* - The DENV-4 strain BR2972/2013, isolated from a patient’s serum, was used in this study. Serotype was identified by real time quantitative polymerase chain reaction (RT-PCR)^47^ and by isolation into *Aedes albopictus* cell line (C6/36 cells; accession number: CRL-1660),^48^ performed by the Laboratório de Flavivirus, Fiocruz. A sample was kindly provided for use in this study.

*Viral stock production* - The viral stock was prepared by inoculating the DENV-4 strain BR2972/2013 into 175 cm^2^ culture cell flasks containing *Ae. albopictus* C6/36 cells^48^ at a concentration of 5x10^5^ cells/mL. Briefly, for virus propagation, *Ae. albopictus* C6/36 cells were grown in Leibovitz medium (L-15, Sigma-Aldrich Corporation , USA) with 10% foetal bovine serum (FBS) (Gibco, Thermo Fisher Scientific Inc., USA) in an incubator at 28ºC. Prior to virus inoculation, L-15 medium was replaced, 2% foetal bovine serum was added and 100 μL of the DENV-4 strain was inoculated and incubated at 28ºC for five days. The virus was harvested by transferring all the flask supernatant to a 15 mL centrifuge tube, centrifuging for 10 min at 4000 x g at 4ºC. Supernatant aliquots were stored at -70ºC for titration. After three cell passages, the strain presented a viral titre of 10^9^ TCID_50_/mL, and was used for experimental infection. The viral titre was calculated using the Reed Muench method.^49^

*Study design* - For this study, thirty mice were used. Fifteen kidney samples were subject to analysis through bright field microscopy, with ten mice being infected with DENV-4 and five mice uninfected, used as mock-infected control. Another fifteen were subject to transmission electron microscopy analysis, following the same aforementioned criteria of ten infected and five uninfected, with one kidney destined to ultrastructural analysis, and the remaining kidney for qRT-PCR analysis.

*Experimental infection* - For experimental infection with DENV-4, two months-old male BALB/c mice, provided by the Instituto de Ciência e Tecnologia em Biomodelos, at Fiocruz, Rio de Janeiro, Brazil, were used. During the experimentation period, the mice were housed in the vivarium of Hélio and Peggy Pereira Pavilion, IOC, Fiocruz (biosafety level 2), and separated in groups of five per cage. Mice were inoculated through the caudal vein with 100 μL of the DENV-4 strain diluted in L-15 medium, which presented a viral titre of 10000 TCID_50_/0.1 mL. For the negative control, mice were inoculated with 100 μL of centrifuged C6/36 cell culture supernatant in L-15 culture medium (Sigma-Aldrich Corporation, USA). All the mice were euthanised 72 h post infection. Euthanasia was performed using a lethal dose of ketamine (150 mg/kg), xylazine (10 mg/kg) and tramadol (10 mg/kg), administered through the intraperitoneal route. Once the anaesthetic effect set in, the mice were subject to cervical dislocation and the organs were harvested.

*Bright field microscopy* - Following the organ harvest, the collected kidneys were placed on a glass plate, and sectioned along the sagittal plane, in two equal halves. Afterwards, the samples were and stored in a histological cassette, and placed in a container containing Millonig’s buffered formalin. Subsequently, the tissue was dehydrated in baths of increasing concentrations of ethanol, clarified in xylene and embedded in paraffin. Tissue sections 5 µm thick were obtained using a using a Leica 2025 microtome (Leica, Germany) and stained with haematoxylin and eosin. Finally, the stained glass slides were analysed on a bright field microscope (AxioHome, Zeiss, Germany).

*Histomorphometry* - Glomeruli count and glomerular area were measured on kidney samples of BALB/c mice. Ten glass slides containing kidney histological sections stained with H&E (Five from mice infected with DENV-4 and five from uninfected mice, of the mock-infected control group) were analysed on a bright field microscope (AxioHome, Zeiss, Germany). For each glass slide, 20 images of random areas were captured at 200 magnification using a coupled camera. For each image, all glomeruli were counted and had their area quantified using the open-source image analysis software ImageJ.

*Statistical analysis* - A database on the glomeruli count and glomerular area of infected and uninfected mice was created in Microsoft Excel and the mean of the values was calculated. The resulting data was analysed using the GraphPad Prism software version 8.0.1 and the SPSS Statistics software version 25. The Shapiro-Wilk test (p > 0.05) was used to assess the normality of data. The Student’s t-test was performed since the data followed a normal distribution and all results of p ≤ 0.05 were considered statistically significant.

*Immunohistochemistry* - Following deparaffinisation and rehydration, the kidney samples underwent antigen retrieval, while submerged in EnVision Flex target retrieval solution, high pH (Dako, USA), inside a pressure cooker. Afterwards, a solution of hydrogen peroxidase in methanol was used, to block endogenous peroxidase. Samples were incubated with either anti-NS3 antibody produced in rabbit (1:200), provided by the Laboratório de Biotecnologia e Bioengenharia Estrutural, of the Universidade Federal do Rio de Janeiro or anti-flavivirus envelope protein antibody produced in mouse (1:200), provided by the Laboratório de Flavivirus, IOC, Fiocruz. Finally, samples were incubated with anti-rabbit antibody horseradish peroxidase conjugate (Advanced Biosystems, USA). Reaction was revealed with diaminobenzidine (Scytek, USA) as chromogen and sections were counterstained with Harris’s haematoxylin (Dako, USA). A reaction control was performed using only the secondary horseradish peroxidase-conjugated antibody.

*Transmission electron microscopy* - Kidney samples were fixated in 3% glutaraldehyde in sodium cacodylate buffer 0.2 M, pH 7.2, stored at 4ºC and processed as described by Barreto-Vieira.^50^ The resulting resin blocks were sliced in ultrathin sections 50-70 nm thick with a Reichert-Jung Ultracut E ultramicrotome (Leica, Germany) and placed on copper grids. These sections were then analysed on a Hitachi HT 7800 transmission electron microscope (Hitachi, Japan).

*Real time quantitative RT-PCR* - For molecular analysis, kidneys were washed with phosphate buffered saline and store at -80ºC. The samples were macerated in L-15 culture medium (Invitrogen, USA) and centrifuged for fifteen minutes at 10000 rpm at 4ºC. Extraction was performed with 140 µL of kidney macerate supernatant, with the QIAmp Viral RNA mini kit (Qiagen, Germany), following the protocol described by the manufacturer. Amplification was performed using the SuperScript III Platinum One-Step Quantitative RT-PCR kit (Invitrogen Corporation, USA) according to the kit’s instructions, using the primers DENJ-4R (5’TCCACCTGAGACTCCTTCCA3’) and DENJ-4F (5’TTGTCCTAATGATGCTGGTCG3’), and probe DENJ-4P (6-FAM 5’TTCCTACTCCTACGCATCGATTCCG3’ BHQ-1).^47^ Reaction was performed in a 7500 Real-Time PCR System (Applied Biosystems, USA).

## RESULTS

**Fig. 1:**
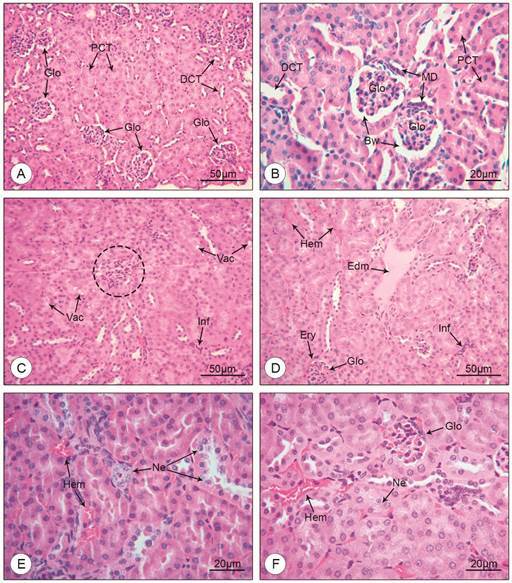
histological sections of BALB/c mice kidney stained with H&E (A, B: uninfected mice; C-F: DENV-4 infected mice). (A, B) Glomerulus (Glo); distal convoluted tubule (DCT); proximal convoluted tubule (PCT); Bowman’s space (Bw); *Macula densa* (MD). (C) Glomerulus presenting reduced Bowman’s space (dashed outline); tubular cells containing cytoplasmic vacuoles (Vac); inflammatory infiltrate (Inf). (D) Areas of interstitial haemorrhage (Hem); capillary oedema (Edm); erythrocytes in the Bowman’s space (Ery) of the glomerulus (Glo); inflammatory infiltrate (Inf). (E, F) Areas of interstitial haemorrhage (Hem); tubular necrosis (Ne); glomerulus (Glo). Magnification: (A, C, D) 200x; (B, E, F) 400x.

**Fig. 2:**
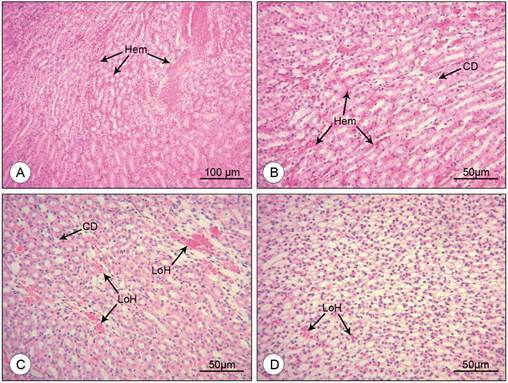
histological sections of DENV-4 infected BALB/c mice kidney stained with H&E. (A, B) Areas of interstitial haemorrhage (Hem); collecting duct (CD). (C, D) Thin part of the loop of Henle containing blood (LoH); collecting duct (CD). Magnification: (A) 100x; (B, C, D) 200x.

*Histopathological, histomorphometrical and ultrastructural alterations* - Mice of the mock-infected control group showed no signs of kidney injury. The glomeruli presented a regular aspect, with normal sized cells and well-defined parietal and visceral layers. Tubular cells also presented no histopathological alterations. Renal tubules had a healthy appearance, with well-preserved lumen and brush border (Fig. 1A-B). Mice infected with DENV-4, on the other hand, showed a noticeable decrease of the area of the Bowman’s Space, due to an apparent increase in the glomerular cellularity (Fig. 1C), oftentimes making it impossible to distinguish the visceral and parietal layers. In some kidney sections where the Bowman’s space was preserved, erythrocytes were observed within, between the layers of the capsule (Fig. 1D). Clear vacuoles were seen in the cytoplasm of tubular cells, some causing the lateralisation of the nucleus (Fig. 1C). The presence of inflammatory infiltrate was discrete, but ubiquitous, represented by small but noticeable clusters of inflammatory cells in the tubular interstice (Fig. 1C-D). Capillary oedema was also observed, although not a common find (Fig. 1D). Distal and proximal convoluted tubules going through different stages of necrosis were present in the tissue (Fig. 1E). Small haemorrhagic foci were present throughout the kidney, both in the medullar and cortical regions (Fig. 1E-F). Larger haemorrhagic areas were present only in the kidney of a mouse that tested positive for DENV-4 envelope protein (Fig. 2A). Blood was present not only in the tubular interstice (Fig. 2B), but also in the lumen of the thin part of the loop of Henle (Fig. 2C-D). The frequency of each histopathological finding is depicted in Table.

**TABLE:** Frequency of histopathological alterations observed in the kidneys of BALB/c mice infected with dengue virus type 4 (DENV-4)

Histopathological alteration	Positive/Tested (%)
Tubular necrosis	9/10 (90)
Inflammatory cell infiltrate	8/10 (80)
Glomerular enlargement	8/10 (80)
Cytoplasmic vacuoles	5/10 (50)
Erythrocytes in the Bowman’s space	5/10 (50)
Haemorrhage	4/10 (40)
Capillary oedema	2/10 (20)

**Fig. 3:**
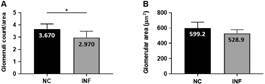
glomeruli count per analysed area (A) and mean area occupied by glomeruli (B) of negative control (NC) and DENV-4 infected (INF) BALB/c mice. *: p < 0.05.

A statistically significant (p = 0.0451) decrease in the glomerular count per analysed kidney was observed in DENV-4 infected mice (Fig. 3A). Glomerular area was also smaller in DENV-4 infected mice, although this alteration was not statistically significant (Fig. 3B).

**Fig. 4:**
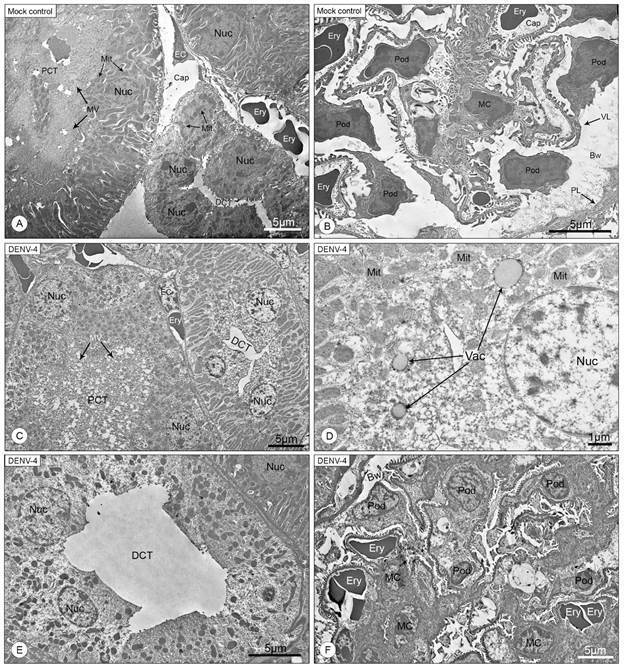
electron micrographs of BALB/c mice kidney sections (A, B: uninfected mice; C-F: DENV-4 infected mice). (A) Proximal convoluted tubule (PCT); microvilli (MV) that form the brush border; nucleus (Nuc); distal convoluted tubule (DCT); mitochondrion (Mit); endothelial cell (EC) that forms the capillary (Cap); erythrocytes (Ery). (B) Podocytes (Pod) that surround the glomerular capillaries (Cap); mesangial cell (MC); visceral layer (VL) and parietal layer (PL) of the Bowman’s space (Bw); erythrocytes (Ery). (C) Cells of the distal convoluted tubule (DCT) presenting loss of cytoplasm (*); nucleus (Nuc); proximal convoluted tubule (PCT) showing loss of microvilli (MV). (D) Vacuoles (Vac) of indistinct origin in the cytoplasm of a distal tubular cell; nucleus (Nuc); mitochondrion (Mit). (E) Cells of the distal convoluted tubule (DCT) presenting loss of cytoplasm (*) and pyknotic nucleus (red dashed outline); nucleus (Nuc). (F) Reduced Bowman’s space (Bw); podocytes (Pod); mesangial cells (MC) presenting expanded mesangial matrix (red arrow); erythrocytes (Ery). Magnification: (A, C) 1.200x; (B, E, F) 1.500x; (D) 2.500x.

**Fig. 5:**
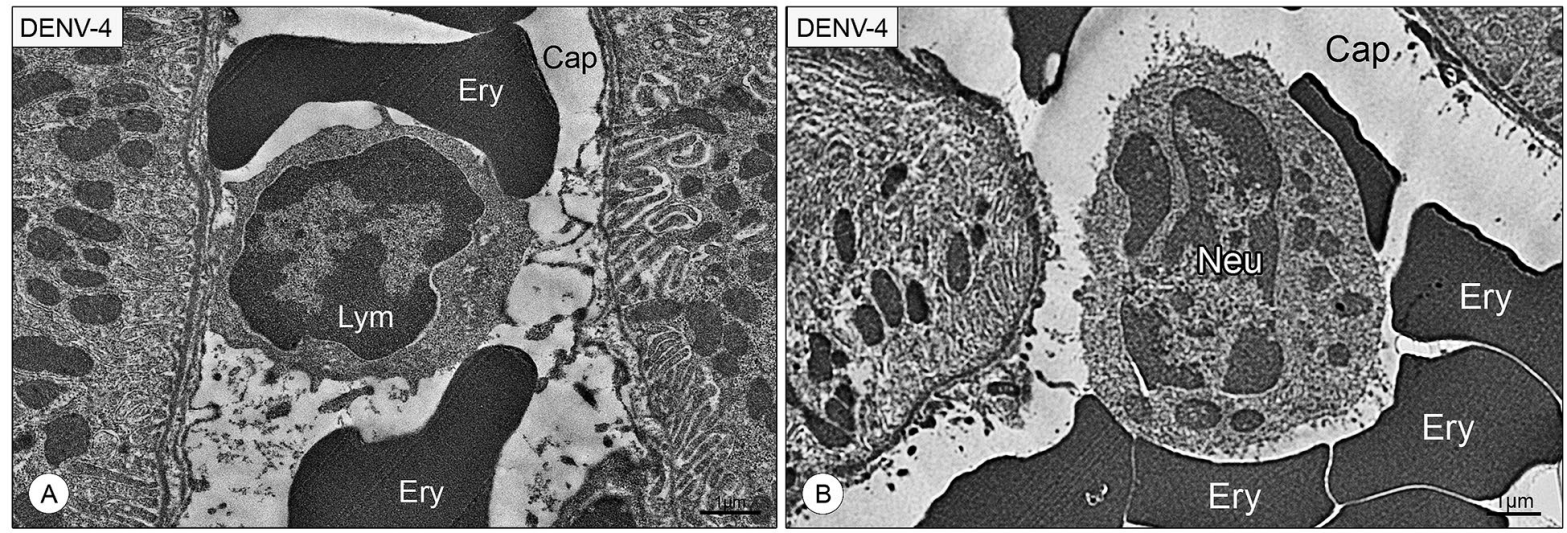
electron micrographs of DENV-4 infected BALB/c mice kidney sections. (A) Lymphocyte (Lym) circulating in a capillary (Cap); erythrocyte (Ery). (B) Neutrophil (Neu) circulating in the capillary; erythrocyte (Ery). (Cap). Magnification: (A) 6.000x (B) 2.000x.

Upon ultrastructural inspection, uninfected kidneys showed no morphological alterations, both in the tubular and in the glomerular structure. Glomerular integrity was well preserved, with a clear distinction of the Bowman’s space and its layers. (Fig. 4A-B). Analysis of the kidneys of mice infected with DENV-4 offered a closer look into the necrotic process of the tubular cells, which presented intense loss of cytoplasm and condensation of the chromatin within the nucleus, characteristic of pyknosis (Fig. 4C, E). Loss of the microvilli that forms the brush border of the proximal convoluted tubule was also observed (Fig. 4C). Vacuoles of unknown origin, smaller than the other described during bright field microscopy analysis, and filled with a substance unlike water or lipids, were observed inside cells of the distal convoluted tubule, more commonly present in the apical region of necrotic cells (Fig. 4D). In the glomeruli, Bowman’s space was reduced, seemingly due to an expansion of the mesangial matrix of mesangial cells. Capillary lumen was also noticeably reduced (Fig. 4F). Inflammatory cells were identified circulating in renal the capillaries and in the tubular interstice, and consisted mostly of lymphocytes (Fig. 5A) and neutrophils (Fig. 5B).

**Fig. 6:**
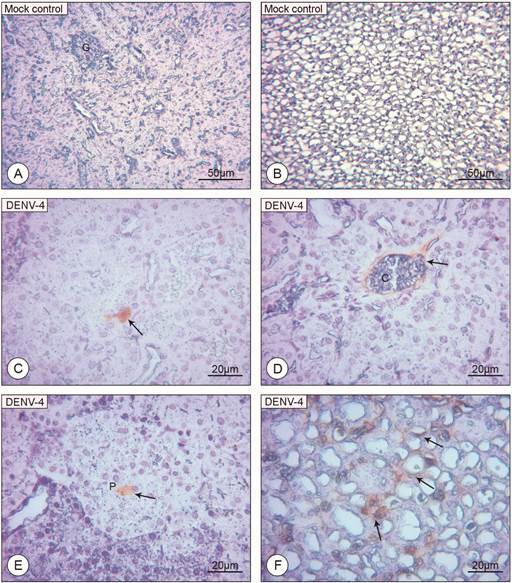
histological sections of BALB/c mice kidney counterstained with Harris Haematoxylin (A, B: uninfected mice; C-F: DENV-4 infected mice) (A, B) Kidney sections showing no peroxidase reactive cells; glomerulus (G). (C) Envelope protein reactive tubular cell (arrow). (D) Envelope protein reactive endothelium (arrow). (E) Envelope protein reactive lumen (arrow) of the proximal convoluted tubule (P). (F) Envelope protein reactive cells of the loop of Henle (arrow). Magnification: (A, B) 200x; (C, D, E, F) 400x.

*Antigen and viral genome detection* - No immunostaining was observed in mice of the control group (Fig. 6A-B). Reaction control also did not show any immunostaining. While the NS3 antigen was not detected in any of the tested kidneys, the envelope protein was detected in the cortical region, in cells of the proximal convoluted tubule (Fig. 6C), in its lumen (Fig. 6E) and in the endothelium of capillaries (Fig. 6D). In the medullar region, immunostaining was observed in cells of the loop of Henle (Fig. 6F). The envelope protein was detected in four out of eight (50%) tested kidney samples. DENV-4 viral RNA was not detected in any of the mice kidneys tested through qRT-PCR.

## DISCUSSION

Overall, the results described in this study are in line with what is seen in human cases of dengue presenting renal manifestations. Histopathological alterations observed fit the descriptions of dengue-induced AKI published in the related literature,^13^ ^,^ ^16^ albeit milder, and non-lethal.

Vascular alterations induced by DENV seem to be the main cause for acute tubular necrosis, due to a decrease in renal perfusion, which in turn leads to ischemia.^51^ Rhabdomyolysis, a muscular manifestation of DENV infection, while seemingly unrelated to kidney injury, also plays a large role in the development of acute tubular necrosis during dengue. This condition is characterised by the necrosis of skeletal muscle cells, resulting in the release of proteins in the circulation. One of these proteins, myoglobin, is known to deposit in the renal tubules, causing tubular injury and tubular obstruction.^11^ ^,^ ^28^ The loss of microvilli described here could represent the initial stages of proximal convoluted tubule necrosis, characterised by loss of the brush border.^31^ Even though some tubular cells appeared to be undergoing necrosis, the process did not seem widespread, and could perhaps be self-limited, reversible even, following the resolution of the disease. The vacuoles observed in tubular cells of the cortical region through bright field microscopy, though remarkable, are not completely unusual, at least in male mice. Even if these vacuoles were not perceived in mice of the control group, their appearance is said to be benign, and not a major histopathological find.^52^ The smaller unidentified vacuoles, however, were only observed through transmission electron microscopy, and could be a direct result of the necrosis of the cells, since they were only seen in necrotic cells, in cellular regions suffering from loss of cytoplasm.

While Póvoa et al.^6^ did not detect the viral NS3 antigen in the kidney of human fatal cases of dengue, nor the presence of viral RNA, they did observe antigens hypothesised to be either the envelope or the membrane proteins in monocytes and macrophages. Jessie et al.^23^ have also detected DENV antigens in tubular cell epithelium, with the absence of viral RNA in the renal tissue. Both authors suggest that this could be due to these different cell types reabsorbing the circulating immune complexes, with the same happening during yellow fever infection.^23^ The deposition of immune complexes has been described in the glomeruli of BALB/c mice^53^ and the presence of glomerular immune complex deposits has been associated to mesangial cell hypertrophy, observed through transmission electron microscopy.^54^ Wiwanitkit^55^ has suggested that, due to the size difference between the dengue virus-immunoglobulin immune complex and the glomerular capillary ― the latter being much larger than the former ― entrapment of the immune complex in the glomerulus should not easily happen, unless there is a significant narrowing of the glomerular diameter, due to lesion or infection.

In this study, although DENV RNA was not detected in kidney, the envelope protein was present in tubular cells and tubular lumen, cells of the loop of Henle and endothelium wall, suggesting that viral antigen reabsorption is likely to happen in the tubular pathway, should immune complexes end up being filtered by the glomerulus. Endothelial cells, on the other hand, are known to play a major role in the pathogenesis of dengue.^56^ Some authors have previously suggested that the envelope protein is capable of modifying the vascular permeability of the endothelium, either directly, altering the morphology of endothelial cells,^57^ or through the infection of monocytes, which, in turn, contribute to viral replication and production of nitric oxide and cytokines, and, consequently, to an increase in endothelial permeability.^58^ Cytokines and chemokines are also known to damage the tissue, being secreted by macrophages and T lymphocytes during the attempt to contain the viral infection.^59^ ^,^ ^60^ The cytokines IL-17 and IL-18, in particular, seem to be widely expressed in the kidney during severe cases of dengue.^19^ In a recent study, Oliveira et al.^61^ successfully detected the NS3 antigen in the kidney of fatal dengue cases in children. The presence of the viral antigen in mesangial and endothelial cells of the glomerulus, and in monocytes and macrophages, suggests that viral infection and replication can occur in the kidney in severe cases of dengue.^61^

Also described in this study was the increased size of the glomeruli, better observed through transmission electron microscopy. This alteration is the result of the expansion of the mesangial matrix of mesangial cells, leading not only to a decrease in the area of the Bowman’s space, through where the glomerular filtrate flows, but also to the compression of the glomerular capillaries, possibly diminishing filtration rates, and facilitating the entrapment of immune complexes. Although the virus was not detected in kidney, the envelope protein was present. This could be a result of the circulation of the virus in the blood. Like Jácome et al.,^46^ we also observed a decrease in glomeruli count in each analysed kidney, suggesting the possibility of glomerular atrophy. Curiously, histomorphometrical data showed an actual decrease in glomerular area. While these results conflict with what was seen during histopathological and ultrastructural analysis, they were not statistically significant. Alterations in the kidney function are also linked to glomerular injury, and are often reported during dengue. These are associated to several biochemical imbalances, such as the increase of blood urea nitrogen and blood creatinine levels, both in BALB/c mice and in humans.^6^ ^,^ ^27^ ^,^ ^41^ ^,^ ^62^

Haemorrhage and vascular leakage are hallmarks of dengue and SD, probably induced by cytokines. TNFα, IL6 and IL8 are known to alter the vascular permeability of capillaries, triggering cases of vascular leakage in dengue.^63^ The reduced blood flow to the kidney likely leads to an ischemic process, and is the reason for the necrosis of tubular cells.^6^ The presence of blood in later portions of the nephron suggests alterations in the kidney filtering capabilities. Furthermore, the increase of vascular permeability induced by DENV infection could also alter the filtration process, facilitating the passage of blood through the glomerular endothelium and onto the renal tubules. The areas of haemorrhage seen in the tubular interstice also indicate the occurrence of these haemodynamic alterations, reinforced by the presence of the viral antigen in the endothelial wall. Although blood was never directly observed in the urine of mice upon clinical inspection, the possibility of microscopic haematuria remains.^64^ Larger haemorrhagic areas in kidneys positive for the envelope protein could indicate a correlation between the presence of the viral antigen and haemorrhage severity. An interesting perspective for a similar study would be the analysis of mice urine through urinalysis, to investigate the presence of proteinuria or haematuria.

The histopathological alterations observed in this study seemed much milder than those observed by Jácome et al.^46^ This could be due to the simple fact that DENV-4 causes a less severe disease, when compared to other DENV serotypes.^44^ Another possibility is that the kidney is not a primary target of dengue infection in mice, at least not under normal circumstances. This is not to say that infection of the kidney does not happen, just that it is less likely during a primary DENV infection. Nonetheless, the inoculation of the virus in the blood is enough to cause damage to the renal tissue, either through the action of cytokines, of circulating immune complexes and/or due to vascular permeability alterations. In the end, as is with all topics surrounding the pathogenesis of dengue, much is yet to be uncovered.
